# Angiogenesis and Related Disorders: Emerging Insights and Future Directions

**DOI:** 10.3390/biomedicines13112730

**Published:** 2025-11-07

**Authors:** Hongmin Yao, Jian Li

**Affiliations:** 1 Institute for Biomedical Sciences, Georgia State University, Atlanta, GA 30303, USA; 2 Emory Transplant Center, Department of Surgery, Emory University, Atlanta, GA 30322, USA

## 1. Introduction

Angiogenesis, the formation of new blood vessels from preexisting vasculature, is fundamental to both physiological homeostasis and pathological remodeling. First described by John Hunter in the late 18th century, this process underlies normal development, wound healing, and tissue adaptation to metabolic demand, such as in skeletal and cardiac muscle during exercise or in adipose tissue expansion during weight gain. However, dysregulated angiogenesis contributes to a wide range of diseases, including cancer, atherosclerosis, diabetic retinopathy, macular degeneration, pulmonary hypertension, and ischemic heart disease.

Over the past five decades, intensive research has established angiogenesis as a pivotal therapeutic target. While pro-angiogenic strategies have been explored to restore perfusion in ischemic tissues and promote wound healing, anti-angiogenic therapies have transformed the management of tumors and ocular neovascular diseases [1]. Indeed, the U.S. Food and Drug Administration has approved more than a dozen anti-angiogenic drugs for metastatic and ophthalmologic indications, underscoring the clinical relevance of vascular modulation [2].

Despite these advances, translating fundamental discoveries into durable clinical success remains a major challenge, largely due to the complexity and heterogeneity of endothelial biology across tissues and diseases. The Special Issue “Angiogenesis and Related Disorders” aims to highlight recent conceptual and technological progress in this field, with particular emphasis on the translational aspects of angiogenesis research. The collection encompasses studies on a range of topics, from molecular characterization of endothelial subtypes to computational modeling, tissue engineering, and clinical implications. Together, these contributions offer new insights into the mechanisms of vascular growth and remodeling, identify potential biomarkers and therapeutic targets, and chart a path toward more precise and personalized interventions.

## 2. Recent Advances in Targeting Angiogenesis Therapy

This Special Issue, “Angiogenesis and Related Disorders,” highlights diverse and translational advances that deepen our understanding of vascular biology and therapeutic targeting.

Mamilos et al. explored the role of nestin, an intermediate filament protein associated with proliferating cells, as a marker to distinguish between blood and lymphatic vessels. They demonstrated that nestin is selectively expressed in endothelial cells of blood vessels but not in lymphangiomas, underscoring its potential as a diagnostic marker [3].

Nivlouei et al. developed a computational hybrid meshless framework that integrates blood flow dynamics for the quantitative assessment of vascular remodeling. This innovative platform enables in silico evaluation of pro- or anti-angiogenic agents prior to in vivo validation, providing a cost- and time-efficient preclinical tool [4].

Li et al. performed a pan-cancer single-cell RNA-seq meta-analysis encompassing 575 patients across 19 solid tumor types. They identified CXCR4^+^ tip cells as endothelial subsets with the highest angiogenic potential, correlating positively with response rates to anti-angiogenic therapies. Conversely, the scarcity of SELE^+^ proinflammatory venous ECs was associated with poor immune infiltration and unfavorable prognosis, suggesting that endothelial heterogeneity influences therapeutic responsiveness [5].

Lee et al. combined a microfluidic organ-on-a-chip angiogenesis model with scRNA-seq to dissect the spatial and temporal dynamics of endothelial subtypes. They discovered distinct autophagic activation patterns in tip and stalk cells, indicating that autophagy exerts cell type-specific regulatory roles during sprouting angiogenesis. These findings suggest that modulating autophagy could become a new layer of therapeutic control in angiogenic disorders [6].

In lung adenocarcinoma, angiogenesis profoundly shapes the tumor microenvironment [7]. Tang et al. identified two angiogenesis-related gene clusters, defining an angiogenesis-related score (ARS) predictive of immune phenotype and treatment outcome. High-ARS tumors exhibited pro-oncogenic immune signatures and poor prognosis, whereas low-ARS tumors were more responsive to immunotherapy, highlighting the prognostic and therapeutic utility of angiogenesis gene profiling [8].

In the cardiovascular field, Yu et al. reported that ANGPT2, an endothelial-derived angiogenic mediator, contributes to intracranial aneurysm (IA) formation and progression. Single-cell transcriptomic profiling of over 43,000 cells from IA patients and healthy aortas revealed ANGPT2-expressing endothelial cells as key pathogenic drivers. Genetic depletion of *angpt2* in zebrafish significantly mitigated arterial dilation, suggesting a causal link between angiogenic activation and aneurysm development [9].

Beyond cancer and vascular diseases, angiogenic phenotypes have also been implicated in fibrotic lung disease. Using a murine silicosis model, Cao et al. identified two endothelial populations, one proinflammatory and one reparative/angiogenic. The latter diminished during disease progression, suggesting impaired endothelial plasticity as a pathogenic mechanism in silicosis [10].

Finally, Chen et al. demonstrated that during heart transplantation, lymphangiogenesis within grafts arises primarily from recipient-derived vessels, driven by fibroblast-secreted VEGF-C. Excessive lymphangiogenesis correlated with graft arteriosclerosis, suggesting that selective inhibition of lymphatic remodeling could improve transplant outcomes [11].

Collectively, these studies illustrate the multifaceted role of angiogenesis in health and disease, spanning computational modeling, single-cell profiling, tissue engineering, and translational therapeutics.

## 3. Challenges and Knowledge Gaps

Though there has been remarkable progress, significant challenges remain when it comes to translating angiogenesis research into effective therapies. The heterogeneity of endothelial cells across organs and disease contexts complicates biomarker identification and limits the universality of therapeutic targets. Although pro-angiogenic strategies, such as VEGF-A administration or gene therapy [12], have been developed, their clinical success has been modest, largely due to difficulties associated with spatially and temporally controlling VEGF bioactivity. Likewise, resistance to anti-angiogenic therapies and poor drug biodistribution, particularly in hypoxic or immune-excluded tumor regions, continue to limit clinical efficacy. The complex interplay between angiogenesis and the immune microenvironment further adds to the challenge, as excessive vascular pruning can hinder immune cell infiltration while abnormal vasculature may promote tumor progression and metastasis. Emerging evidence suggests that combination therapies, such as dual angiogenic inhibition [13], anti-angiogenesis with immune checkpoint blockade [14], or synergy with chemotherapy [15], hold promise for overcoming these limitations, yet the rational design, patient stratification, and long-term safety of such approaches require deeper investigation.

## 4. Future Perspectives and Research Directions

Future angiogenesis research will likely advance through integrative and technology-driven strategies. The incorporation of single-cell and spatial multi-omics approaches will refine our understanding of endothelial plasticity and cell–cell communication in both physiological and pathological contexts, paving the way for the discovery of context-specific angiogenic regulators. Dynamic imaging and organ-on-chip technologies should be further developed to visualize real-time endothelial behaviors and drug responses, bridging the translational gap between in vitro models and in vivo physiology. Increasing attention should also be given to endothelial metabolism and autophagy, as these processes critically modulate angiogenic potential beyond canonical VEGF signaling. Furthermore, a deeper understanding of immune–angiogenic crosstalk will be essential for optimizing combination therapies that simultaneously target tumor vasculature and immune activation.

Precision and personalization will define the next generation of angiogenesis-targeted treatments. Artificial intelligence-based predictive modeling and biomarker-guided patient selection may enhance therapeutic efficacy and minimize resistance. Additionally, the often-overlooked roles of lymphangiogenesis and vascular remodeling in disease progression, such as in transplantation, cardiovascular disorders, and metastasis, represent a promising and underexplored frontier. Through these avenues, future research in angiogenesis is expected to move toward integrative, patient-specific, and mechanism-based interventions that will ultimately transform basic vascular biology into precision medicine.
